# Vestibular Disorders Limited to the Vertical Semicircular Canals

**DOI:** 10.1055/s-0044-1786047

**Published:** 2024-05-25

**Authors:** Pedro Luiz Mangabeira Albernaz, Flavia Salvaterra Cusin, Bernardo Faria Ramos, Renato Cal, Francisco Carlos Zuma e Maia

**Affiliations:** 1 Department of Otolaryngology, Hospital Israelita Albert Einstein, São Paulo, SP, Brazil; 2 Department of Audiology, Hospital Israelita Albert Einstein, São Paulo, SP, Brazil; 3 Department of Otolaryngology, Faculdade de Medicina, Universidade Federal do Espírito Santo, Vitória, ES, Brazil; 4 Department of Otolaryngology, Faculdade de Medicina, Instituto de Ciências Médicas, Universidade Federal do Pará, Belém, PA, Brazil; 5 Department of Surgery, Faculdade de Medicina, Universidade Federal do Rio Grande do Sul, Porto Alegre, RS, Brazil

**Keywords:** vestibular diseases, semicircular canals, head impulse test

## Abstract

**Introduction** The advent of the video head impulse test (vHIT) enables the study of each one of the six semicircular canals. In the present study, certain patients present disorders related only to the vertical semicircular canals, and they were carefully evaluated.

**Objective** To investigate vestibular disorders limited to the vertical semicircular canals.

**Methods** In total, 9,891 patients were submitted to the vHIT in our clinic; 26 (2.63%) of them, 11 men and 15 women, showed reduced vestibulo-ocular reflex (VOR) limited to the vertical canals. All of these patients had vestibular symptoms.

**Results** These patients presented different disorders of the vestibular system, and ten of them presented vestibular neuritis.

**Conclusion** Now, vestibular disorders limited the vertical canals can be evaluated through the vHIT. These disorders, however, may relate do different labyrinthine diseases.

## Introduction

In the last few years, the video head impulse test (vHIT) has become an important additional examination in neurotology. Actually, it became a significant part of the functional evaluation of the vestibular system. 1

For many years, caloric tests were the only way to stimulate each ear separately. Caloric tests are not physiological; the vestibular system is related to movements and accelerations, which are quite different from thermal convection currents affecting the lateral semicircular canals. The superior and posterior canals could not be investigated.

The vHIT, however, can test the six semicircular canals, and it has been used in our clinics according to the standard technique. 2 MacDougall et al. 3 established the importance of the vHIT test for the diagnosis of disorders of the vertical canals.

The current study consists in an evaluation of 26 patients who presented vestibular changes limited to the vertical semicircular canals in the vHIT test.

## Methods

Between January 2013 and March 2023, 9,891 patients were submitted to the vHIT test in our clinic. Most of these patients presented reduced vestibulo-ocular reflex (VOR) in one or several semicircular canals. For 26 of them, only the anterior and/or posterior semicircular canal presented changes. The percentage of cases with altered VOR responses limited to the vertical canals was of 2.63%, meaning that this pathology is relatively uncommon.

**Fig. 1:**
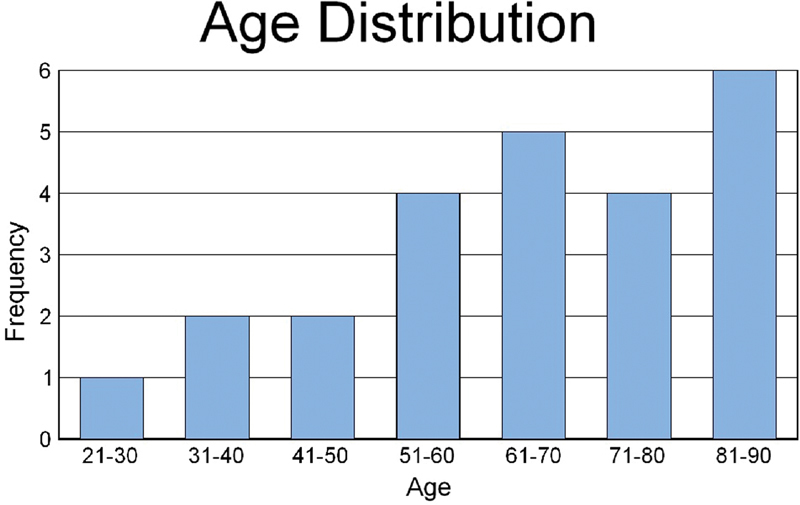
Age distribution.

The group consisted of 11 men and 15 women with ages ranging from 47 to 90 years. Fig. 1 shows the age distribution. All the patients were submitted to an evaluation that included a detailed clinical history and otolaryngological examination. Audiological tests were also performed, including pure-tone audiometry, speech discrimination tests, and immittance tests. Vestibular tests were performed, including spontaneous and gaze nystagmus, positional nystagmus, pendular eye tracking, optokinetic nystagmus, pendular rotatory tests, and, in some cases, caloric tests.

A diagnosis for each of the patients was established through their clinical histories, vestibular tests, and, in some cases, magnetic resonance imaging (MRI) scans. The vHIT tests were performed with the ICS Impulse system (Otometrics/Natus Medical Incorporated, Taastrup, Copenhagen, Denmark). Recordings were obtained for each of the six semicircular canals in all patients. The present research was approved by the Ethics Committee of Hospital Israelita Albert Einstein (CAAE: 06137012.3.0000.0071).

## Results

The vestibular tests were essentially normal, except among the patients with central disorders, who presented alterations of the spontaneous nystagmus and/or gaze nystagmus.

Table 1 shows the diagnoses of these 26 patients, based on their clinical histories and findings of the vestibular examinations. Table 2 shows the semicircular canals that presented altered VOR.

**Table 1:** Diagnoses of 26 patients

DIAGNOSIS	N
Vestibular neuritis	10
Benign paroxysmal positional vertigo	3
Vestibular schwannoma	2
Central vestibular syndrome	2
Menière disease	1
Bacterial labyrinthitis	1
Cranial trauma	1
Vestibular migraine	1
Facial paralysis	1
Orthostatic hypotension	1

**Table 2:** Semicircular Canals with altered vestibulo-ocular reflex (VOR)

SEMICIRCULAR CANALS	N
Left posterior	13
Right posterior	5
Right anterior	3
Left anterior + left posterior	2
Left posterior + right posterior	2
Left anterior + right anterior	1
Left anterior	0

Two of ten patients with vestibular neuritis had had previous falls, and one had intense tinnitus. Four of the ten patients had altered VOR of the left posterior canal, and three, of the right posterior canal. In one case, there was reduced response of the left anterior and right anterior canals. One patient presented reduced response of the left anterior and posterior canals.

Of the three patients with vestibular paroxysmal positional vertigo, one presented reduced response of the left posterior canal, another, reduced response of the right anterior canal and the third presented reduced responses of the left anterior and posterior canals.

The two patients with vestibular schwannoma on the left side presented reduced VOR of the left posterior canal. Of the two cases of central vestibular syndrome, one presented changes in the right anterior canal and another, in the left and right posterior canals. The patient with Menière disease presented reduced VOR in the right posterior canal. The patient with bacterial labyrinthitis presented reduced VOR in the left posterior canal. The one that suffered a cranial trauma presented reduced response of the right posterior canal. The patient with vestibular migraine presented reduced VOR in the left posterior canal. The patient with left facial paralysis presented a reduced response in the left posterior canal. The case of orthostatic hypotension presented a lower VOR in the left posterior canal.

Overt saccades were observed in one of the patients with vestibular neuritis, one with facial paralysis and one with unsteadiness. Overt and covert saccades were observed in a patient presenting vestibular neuritis and intense tinnitus.

In addition to the patients that had a diagnosis established, there were three patients with unsteadiness. One had had falls and one had a simultaneous episode of presyncope. Two of these patients had reduced VOR in the left posterior canal, another, reduced VOR in the left anterior and posterior canals.

**Fig. 2:**
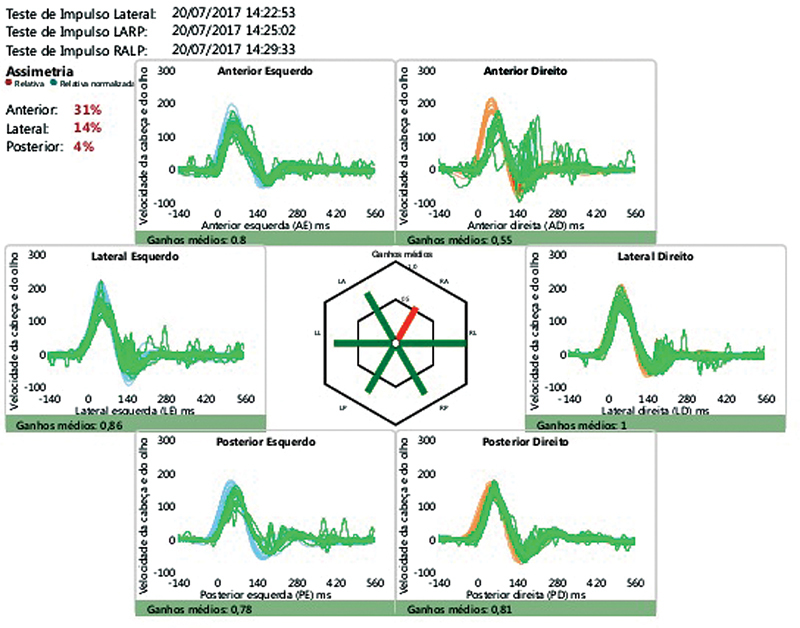
Video head impulse test showing dysfunction in the right anterior semicircular canal.

**Fig. 3:**
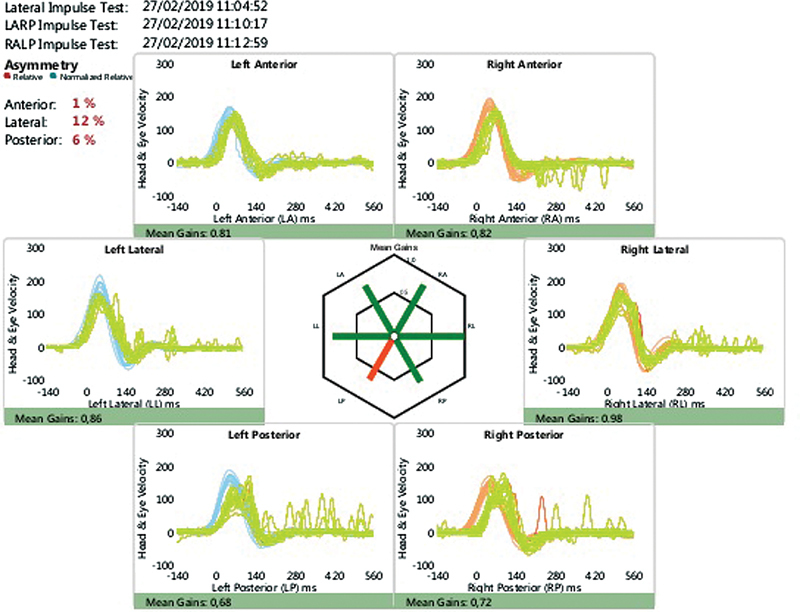
Video head impulse test showing dysfunction in the left posterior semicircular canal.

**Fig. 4:**
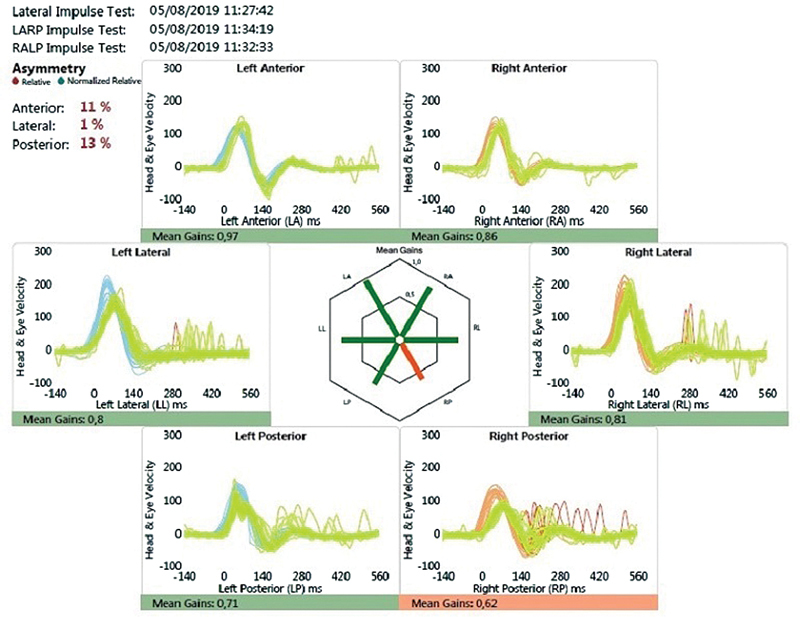
Video head impulse test showing dysfunction in the right posterior semicircular canal.

Figs. 2 , 3 and 4 show the responses of a few patients.

## Discussion

No relationship could be established between the responses of the vertical semicircular canals and the diagnoses of these patients.

There is a significant possibility that some of the responses of the vertical canals may present artifacts. Several patients (four with benign paroxysmal positional vertigo [BPPV], one with a vestibular schwannoma, one with vestibular neuritis, 1 with facial paralysis, one with Menière disease, and 1 with unsteadiness) presented low gain responses without refixation saccades, which would be expected in these cases. In some patients (two with vestibular neuritis, one with BPPV, and one with unsteadiness), the stimulation employed smaller speeds; this happened in some of our early examinations, following a study by Barin, 4 who established that the optimal head velocities for the vertical canals range from 50° to 200° per second. On the other hand, all of these patients had vestibular symptoms and presented normal responses of the lateral canals.

Vestibular neuritis affecting the vertical canals has been reported by Kim and Kim, 5 Alzuphar and Maire, 6 Halmagyi et al., 7 Taylor et al., 8 and Nayak et al. 9 It is important, however, to know that other pathologies may also affect the vertical canals.

Imbaud-Genieys 10 described a series of 20 patients with anterior semicircular canal BPPV. Only one out of our patients presented reduced VOR limited to the anterior canal.

Fujiwara et al. 11 studied the function of the vertical canal in patients with vestibular schwannoma, and found that dysfunction in the anterior and posterior semicircular canals was detected by the vHIT in 26.7% and 60.0% of the patients respectively. Fukushima et al. 12 described vertical canal dysfunction in a patient with early-stage Menière disease. Salmito and Ganança 13 studied the vHIT in patients with vestibular migraine. No reports were found on unsteadiness, central vestibular syndromes, bacterial labyrinthitis, cranial trauma, facial paralysis, or orthostatic hypotension.

Disorders of the vertical semicircular canals may appear in many situations, and it is important to realize that the patients with this type of vestibular disorder could only be examined by the vHIT.

## Conclusion

Vestibular disorders restricted to the vertical semicircular canals can now be evaluated by the vHIT. All of these patients presented normal responses for the lateral semicircular canals as well as vestibular symptoms. These disorders, however, may relate to several labyrinthine diseases. The vHIT results show many variations and do not lead to the diagnosis of these disorders.
